# Norfluoxetine Prevents Degeneration of Dopamine Neurons by Inhibiting Microglia-Derived Oxidative Stress in an MPTP Mouse Model of Parkinson's Disease

**DOI:** 10.1155/2018/4591289

**Published:** 2018-12-30

**Authors:** Kyung In Kim, Young Cheul Chung, Byung Kwan Jin

**Affiliations:** ^1^Department of Neuroscience, Graduate School, Kyung Hee University, Seoul 02447, Republic of Korea; ^2^Department of Biochemistry and Molecular Biology, School of Medicine, Kyung Hee University, Seoul 02447, Republic of Korea

## Abstract

Neuroinflammation is the neuropathological feature of Parkinson's disease (PD) and causes microglial activation and activated microglia-derived oxidative stress in the PD patients and PD animal models, resulting in neurodegeneration. The present study examined whether norfluoxetine (a metabolite of fluoxetine) could regulate neuroinflammation in the 1-methyl-4-phenyl-1,2,3,6-tetrahydropypridine (MPTP) mouse model of PD and rescue dopamine neurons. Analysis by tyrosine hydroxylase (TH) immunohistochemistry demonstrated that norfluoxetine prevents degeneration of nigrostriatal dopamine neurons *in vivo* in MPTP-lesioned mice compared to vehicle-treated MPTP-lesioned control mice. MAC-1 immunostaining and hydroethidine histochemical staining showed that norfluoxetine neuroprotection is accompanied by inhibiting MPTP-induced microglial activation and activated microglia-derived reactive oxygen species production *in vivo*, respectively. In the separate experiments, treatment with norfluoxetine inhibited NADPH oxidase activation and nitrate production in LPS-treated cortical microglial cultures *in vitro*. Collectively, these *in vivo* and *in vitro* results suggest that norfluoxetine could be employed as a novel therapeutic agent for treating PD, which is associated with neuroinflammation and microglia-derived oxidative stress.

## 1. Introduction

Parkinson's disease (PD) is one of the most common neurodegenerative disorders associated with loss of dopamine (DA) in striatum and death of DA neurons in substantia nigra (SN) [1]. PD neuropathogenesis is closely associated with microglial activation and oxidative stress, which may cause chronic neurodegeneration through reactive oxygen species (ROS) and/or reactive nitrogen species (RNS) [2, 3]. Activated microglia-derived oxidative stress is observed in the SN of PD patients and in the SN of MPTP-treated mouse and lipopolysaccharide- (LPS-) treated rat, which contributes to degeneration of DA neuron [4–7]. Moreover, ROS and/or RNS generating enzyme, such as NADPH oxidase and inducible nitric oxide synthase (iNOS), are upregulated in the SN of PD patients and in the SN of MPTP-treated mouse and LPS-treated rat [6, 7].

Antidepressant has been increasingly suggested as a neuroprotective agent by regulating intrinsic immune mechanism in the brain [8, 9]. Classical selective serotonin reuptake inhibitors (SSRIs) such as fluoxetine, imipramine, and paroxetine have shown an anti-inflammatory effect on LPS-treated microglial cells *in vitro* and *in vivo* [10–12]. Many preclinical and experimental studies reported that fluoxetine has a neuroprotective effect on ischemic stroke [13, 14] and spinal cord injury [15] via downregulating neuroinflammatory molecules. Fluoxetine prevents degeneration of midbrain DA neurons in MPTP-lesioned mice through inhibiting activated microglia-derived oxidative damage and inflammatory response *in vivo* apart from the effect on serotonin uptake [5]. Therefore, understanding the property of fluoxetine and its derivatives is applicable to develop the new therapeutic agents to treat PD patients.

Norfluoxetine is a pharmacologically active metabolite of fluoxetine [16, 17] and formed by N-demethylation of fluoxetine in the liver [18]. Norfluoxetine has a similar potency and selectivity of fluoxetine for inhibition of serotonin reuptake in the brain [17, 18]. Although norfluoxetine is known to permeate the blood-brain barrier and has a higher half-life than fluoxetine [16, 19], its neuroprotective property has not been studied in neurodegenerative disease model. In the present study, we examined whether norfluoxetine could decrease microglial activation and activated microglia-derived ROS production in the SN of MPTP-treated mice *in vivo*, resulting in the survival of DA neurons. In the separate experiments, we investigated whether norfluoxetine could inhibit NADPH oxidase activation and RNS production in LPS-treated cultures of rat microglia *in vitro*.

## 2. Materials and Method

### 2.1. MPTP Intoxication and Drug Treatment

All experiments were conducted in accordance with the approved animal protocols and guidelines established by the Animal Care Committee at Kyung Hee University. Adult male C57BL/6 mice (8- to 10-week-old) were obtained from Daehan Biolink (Eumseong, Korea). All animals were allowed to acclimate to their new surrounding at least 7 days and maintained in a room 20-22°C on a 12-hour light/dark cycle with food and water available ad libitum before PBS and MPTP treatment. As previously described (Chung, 2010 #1), mice were used for MPTP intoxication. MPTP (20 mg kg^−1^, free base; Sigma) was dissolved in PBS and then intraperitoneally injected into mice for four times at 2-hour intervals. For norfluoxetine treatment, 1, 5, and 10 mg kg^−1^ of the norfluoxetine (Sigma) were intraperitoneally administered for 12 hours starting from the last MPTP injection.

### 2.2. Immunohistochemistry

Brains were removed from the skull, postfixed overnight in a buffered 4% paraformaldehyde at 4°C, stored in a 30% sucrose solution for 24 to 48 hours at 4°C until they sank, and were frozen-sectioned on a sliding microtome in 30 *μ*m thick coronal sections. All sections were collected in six separate series and processed for immunohistochemistry. Briefly, the sections were rinsed in 0.1 M PBS and labeled with primary antibodies (rabbit antityrosine hydroxylase (TH; 1 : 2000; Pel-Freez, Brown Deer, WI, USA), rat antimacrophage Ag complex-1 (MAC-1; 1 : 200; Serotec, Oxford, UK)). After overnight incubation, the sections were washed and incubated with proper biotinylated secondary antibodies for 1 h. After washing, the sections were stained with an ABC kit for 1 hour at room temperature, according to the manufacturer's instructions. After washing in PB, the sections were incubated in 0.05% 3,3′-diaminobenzidine (DAB; Sigma) in 0.1 M PB containing 0.003% H2O2. Stained samples were washed in PB and mounted on gelatin-coated slides and analyzed under a bright-field microscope (Nikon, Tokyo, Japan).

### 2.3. Stereological Cell Counting

To determine the total number of TH-positive cells in SN, unbiased stereological cell counting were used. As previously described [4, 5], every 6th section from the entire SN from the rostral tip of the pars compacta back to the caudal end of the pars reticulate (anterioposterior, −2.06 to −4.16 mm from the bregma) was collected, and a total of 6-7 sections/animal are immunostained and counted within a counting grid (150 × 150 *μ*m). Actual counting was performed using a ×100 oil immersion objective.

### 2.4. Densitometric Analysis

Densitometric analyses of the mouse striatum were performed as described previously (Chung, 2010 #1). 17 coronal sections of the striatum were examined at a 5x magnification using the IMAGE-PRO PLUS system (version 4.0, Media Cybernetics, Silver Spring, Maryland, USA) on a computer attached to a light microscope (Zeiss Axioskop, Oberkochen, Germany) interfaced with a CCD video camera (Kodak MegaPlus model 1.4 I, New York, NY, USA). The average of all sections of TH immune-reactivity was calculated separately and statistically processed.

### 2.5. In Situ Detection of O_2_^−^ and O_2_-Derived Oxidants

To detect O_2_^−^ and O_2_-derived oxidants in vivo, 100 *μ*l of hydroethidine (Molecular Probes; 1 mg/ml in PBS containing 1% dimethyl sulfoxide) was administered intraperitoneally for 3 days from the last MPTP injection. After 15 min, the animals were transcardially perfused with a saline solution containing 0.5% sodium nitrate and heparin (10 U/ml) and then fixed with 4% paraformaldehyde in 0.1 M phosphate buffer. After fixation, the brains were cut into 30 *μ*m slices using a sliding microtome. The oxidized hydroethidine product, ethidium, was examined by confocal microscopy (Carl-Zeiss).

### 2.6. Primary Cortical Microglial Culture and Drug Treatment

As previously described [20, 21], cortical microglia were prepared from the cerebral cortices of 1-day-old Sprague–Dawley rats. Briefly, cerebral cortices were triturated into a single cell suspension in a Minimal Essential Medium (MEM) containing 10% FBS and incubated for 2 weeks. Microglia were then detached from 75T-flasks by mild shaking and filtered through a nylon mesh to remove astrocytes and clumped cells. Cultured rat microglia were plated in 24-well plates (5 × 10^4^ cells/well) or 35 mm culture dishes (5 × 10^5^ cells/dish). One hour later, the culture medium was changed to MEM medium containing 5% FBS. At 24 hours after plating, the cells were treated with vehicle, LPS, fluoxetine (Sigma), and norfluoxetine.

### 2.7. NO Measurement and Western Blotting

At 24 hours from LPS treatment, culture media (50 *μ*l) were collected and mixed with the same volume of Greiss reagent (0.1% naphthylethylene diamine, 1% sulfanilamide, and 2.5% H_3_PO_4_). After incubation at room temperature for 1 hour, optical density was measured at 450 nm. Cells were washed with PBS and then homogenized with ProteioExtract™ Native Membrane Protein Extraction Kit (Calbiochem, La Jola, CA, USA) for separating the cytosolic and membrane fractionation. As previously described [4, 5], protein samples were separated with SDS PAGE and transferred to a membrane. The membranes were then incubated overnight at 4°C with rabbit anti-p47phox (1 : 500; Santa Cruz Biotechnology, CA, USA). After washing, the membranes were incubated for 1 hour at room temperature with secondary antibodies (1 : 2000; Amersham Biosciences, Arlington Heights, IL) and washed again. Finally, the blots were developed with enhanced chemiluminescence detection reagents (Amersham Biosciences). The blots were reprobed with antibodies against actin (1 : 2000; Santa Cruz Biotechnology, CA, USA). To determine the relative degree of membrane purification, the membrane fraction was subjected to immunoblotting for calnexin, a membrane marker, using a rabbit polyclonal antibody against calnexin (1 : 1000; Stressgen, British Columbia, Canada). For semiquantitative analyses, the densities of bands on immunoblots were measured with the computer imaging device and accompanying software (Fujifilm).

### 2.8. Statistical Analysis

All data are represented as the means ± SEM. Statistical significance (*p* < 0.05 for all analyses) was assessed using analysis of variance (Instat 3.05, GraphPad, San Diego, CA, USA) followed by Student–Newman–Keuls analyses.

## 3. Results

### 3.1. Norfluoxetine Protects Degeneration of DA Neurons from MPTP Neurotoxicity *In Vivo*

**Figure 1:**
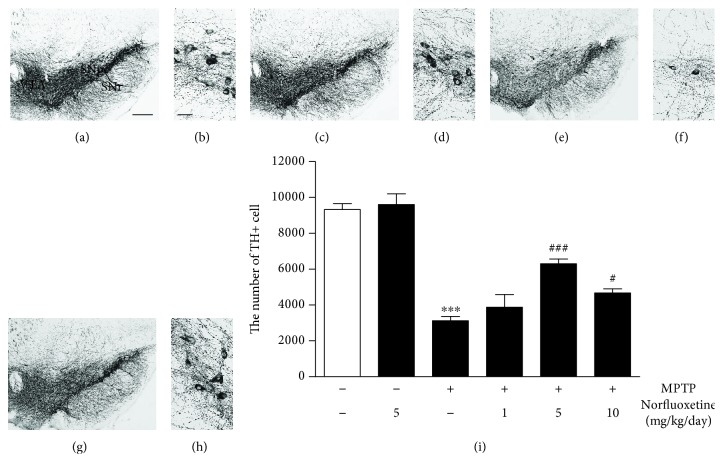
Norfluoxetine prevents degeneration of dopamine neurons in the substantia nigra of MPTP-treated mouse. Animals that received PBS as a control (a, b), norfluoxetine (5 mg/kg) alone (c, d), MPTP (e, f), and MPTP and norfluoxetine (g, h) were sacrificed 7 days after the last MPTP injection. SN tissues were processed for immunostaining with a tyrosine hydroxylase (TH) antibody for dopamine (DA) neurons. (b, d, f, h), higher magnification of (a), (c), (e), and (g), respectively. (i) The numbers of TH^+^ cells in the SNpc. Bars represent the means ± SEM of four to six animals per group. ^∗∗∗^*p* < 0.001 significantly different from control; ^#^*p* < 0.05, ^###^*p* < 0.001 significantly different from MPTP. SNpc: substantia nigra pars compacta; SNr: substantia nigra reticulata; VTA: ventral tegmental area; Con: control; NF: norfluoxetine. Scale bars: 300 *μ*m (left panel) and 50 *μ*m (right panel).

To explore the potential function of norfluoxetine in Parkinson's disease, the mouse MPTP-lesion model of Parkinson's disease was used [4]. The mice received four intraperitoneal injections of MPTP (20 mg/kg) or PBS as a control at 2-hour intervals. Seven days later, brains were processed for immunostaining for tyrosine hydroxylase (TH) to detect DA neurons. Consistent with our previous study [4], analysis by TH immunostaining revealed a significant loss of TH^+^ cells (Figures 1(e) and 1(f)) at 7 days in the MPTP-injected SN compared with PBS-treated controls (Figures 1(a) and 1(b)). When TH^+^ cells in the substantia nigra (SN) were quantified by stereological counts, MPTP treatment attenuated the number of TH^+^ neurons by 67% (Figure 1(i); *p* < 0.001) as compared with the PBS-treated control. Treatment with 1 mg/kg norfluoxetine had a little effect (Figure 1(i)).

**Figure 2:**
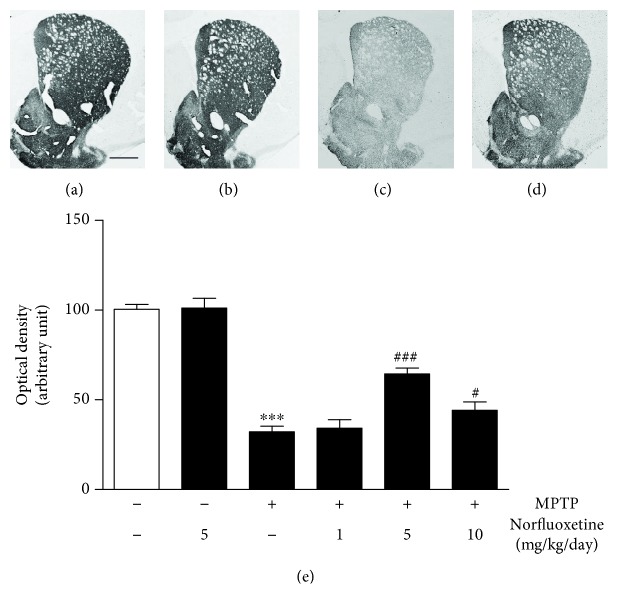
Norfluoxetine prevents degeneration of striatal dopamine fibers in MPTP-treated mouse. The striatal tissues obtained from the same animals as used in Figure 2 were immunostained with a TH antibody for dopamine (DA) fibers. (a) Control. (b) Norfluoxetine. (c) MPTP. (d) MPTP and Norfluoxetine. Scale bar: 650 *μ*m. (e) The optical density of the TH-positive fibers in the STR. ^∗∗∗^*p* < 0.001 significantly different from control; ^#^*p* < 0.05, ^###^*p* < 0.001 significantly different from MPTP. CON: control; NF: norfluoxetine.

Similar to degeneration of TH^+^ cell bodies in the SN, there was a considerable loss of TH^+^ fibers in the striatum (STR; Figure 2(c)) at 7 days in MPTP-injected mice compared with PBS-treated controls (Figure 2(a)). The optical density was quantified by densitometric analysis. MPTP treatment reduced the optical density of TH^+^ fibers by 68% (Figure 2(e); *p* < 0.001) in the STR as compared with the PBS-treated control. Treatment with 1 mg/kg norfluoxetine had a little effect (Figure 2(e)).

To investigate whether norfluoxetine could protect DA neurons from MPTP neurotoxicity, norfluoxetine (1, 5, and 10 mg/kg) was administered for 7 days, starting 12 hours after the last MPTP injection. We chose a 12-hour time point because at this time point, MPTP is fully converted into MPP^+^ and norfluoxetine is unable to interfere MPP^+^ release from astrocytes or uptake of MPP^+^ into DA neurons [5]. The results of TH immunohistochemistry showed that norfluoxetine treatment effectively lessened MPTP-induced loss of DA neurons in the SN (Figures 1(g) and 1(h)) compared to vehicle-treated MPTP-lesioned SN (Figures 1(e) and 1(f)). When quantified and expressed as the percentage of TH^+^ neurons on the SN, administration of 5 or 10 mg/kg norfluoxetine was found to increase the number of TH^+^ neurons by 88% (Figure 1(i); *p* < 0.001) and 48% (Figure 1(i); *p* < 0.05), respectively, compared to vehicle-treated MPTP-injected SN.

Similar to neuroprotection in the SN, treatment with 5 or 10 mg/kg norfluoxetine significantly increased the optical density of TH^+^ DA fibers by 64% (Figure 2(e); *p* < 0.001) and 44% (Figure 2(e); *p* < 0.05) in the STR, respectively, compared to vehicle-treated MPTP-lesioned striatum. When animals received norfluoxetine alone (5 or 10 mg/kg), no substantial reduction of nigral dopamine neurons (Figures 1(c), 1(d), and 1(i)) and their fibers in the STR (Figures 2(b) and 2(e)) was evident in the SN.

### 3.2. Norfluoxetine Inhibits Microglial Activation in MPTP-Treated SN *In Vivo*

**Figure 3:**
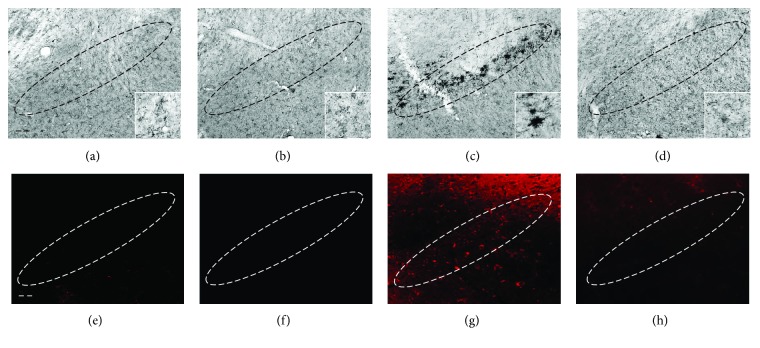
Norfluoxetine decreases MPTP-induced microglial activation and oxidant production in the substantia nigra in vivo. Animals receiving PBS as a control (a, e), norfluoxetine (5 mg/kg per day) alone (b, f), MPTP (c, g), and MPTP + norfluoxetine (d, h) were sacrificed 3 days after the last MPTP injection. Brain tissues were cut and substantia nigra (SN) was immunostained with an antibody for MAC-1 to label microglia (a-d) and prepared for hydroethidine histochemistry to detect oxidant production (e-h). Insets show higher magnifications of (a-d), respectively. Five to seven animals were used for each experimental group. Dotted lines indicate the SNpc. Scale bar: 300 *μ*m.

Activated microglia contribute to loss of midbrain DA neurons in the MPTP model [2, 22]. Thus, we next examined whether the observed neuroprotective effects of norfluoxetine resulted from inhibition of MPTP-induced microglial activation. For this purpose, norfluoxetine (5 mg/kg) was administered for 3 days, starting 12 hours after the last MPTP injection, and brain sections were prepared for immunostaining with MAC-1 to detected microglia. In contrast to the SN sample treated with PBS (Figure 3(a)), where relatively few of MAC-1^+^ cells were seen, MPTP-treated SN samples showed a large number of MAC-1^+^ cells (Figure 3(c)). By contrast, treatment with 5 mg/kg norfluoxetine was found to profoundly lessen the number of MAC-1^+^ cells in the MPTP-injected SN (Figure 3(d)). Treatment with norfluoxetine alone (5 mg/kg) had no effects (Figure 3(b)).

### 3.3. Norfluoxetine Inhibits Microglial NADPH Oxidase-Derived Oxidative Stress in the MPTP-Treated SN *In Vivo*

MPTP was found to induce the production of hydrogen peroxide and O_2_^−^ via NADPH oxidase [4, 7, 23], and O_2_^−^ originating from microglia can produce degeneration of DA neurons in the MPTP-treated SN [5, 24]. Thus, we examined whether MPTP induced O_2_^−^ production in the SN and whether norfluoxetine prevented loss of DA neurons by inhibiting MPTP-induced production of O_2_^−^. To test this, hydroethidine histochemistry was performed on sections adjacent to those used for MAC-1 immunostaining (Figures 3(a)–3(d)) for in situ visualization of MPTP-induced O_2_^−^ production [23]. The fluorescent products of oxidized hydroehtidine (i.e., ethidium accumulation) were significantly increased in the SN 3 days after the last MPTP injection (Figure 3(g)) compared with the PBS-injected SN control (Figure 3(e)). By contrast, MPTP-induced O_2_^−^ was significantly attenuated in SN samples treated with norfluoxetine (5 mg/kg) (Figure 3(h)). These observations are consistent with the data obtained from the MAC-1 immunostaining (Figure 3(c)). Treatment with norfluoxetine alone (5 mg/kg) had no effects (Figure 3(f)).

**Figure 4:**
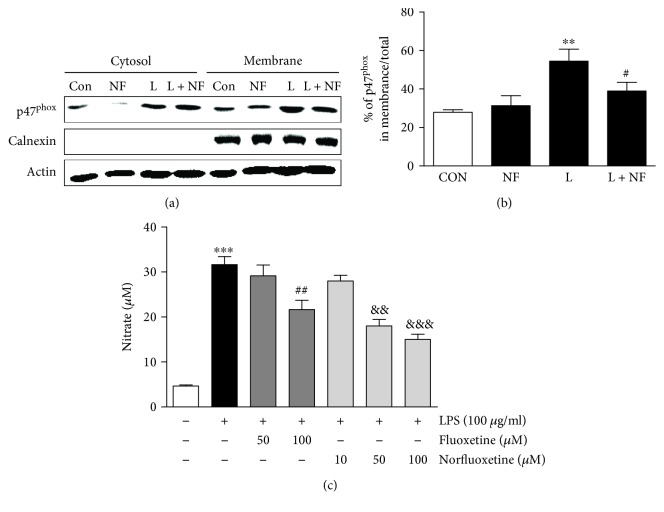
Norfluoxetine attenuates LPS-induced activation of NADPH oxidase and NO production in cortical microglial cultures. Cortical cultures of rat microglia were pretreated with fluoxetine (50 and 100 *μ*M) or norfluoxetine (50 *μ*M) for 6 hr followed by exposure to LPS (100 *μ*g/ml) or vehicle as a control or for 24 h. Cultured cells were homogenized, fractionated, and analyzed NADPH oxidase by Western blot analysis (a, b), and media from cultures were collected for measuring the levels of nitrate (c). (a, b) Western blot analysis showing p47^phox^ activation. The levels of p47^phox^ (NADPH oxidase subunits) in the cytosol and membrane were determined by normalizing calnexin as membrane protein level. CON: control; NF: norfluoxetine; L: LPS; L + NF: LPS and norfluoxetine. Values are expressed as a percentage of the total level of p47^phox^. ^∗∗^*p* < 0.001, compared with nontreated control; ^#^*p* < 0.05, compared with LPS only treated. (c) Nitrate levels are presented as means ± SEM of triplicate cultures on two separate plates. ^∗∗∗^*p* < 0.001, compared with nontreated control cultures; ^##^*p* < 0.01, ^&&^*p* < 0.01, and ^&&&^*p* < 0.001, compared with LPS-only-treated cultures (ANOVA and Student–Newman–Keuls analyses).

As NADPH oxidase is the source of O_2_^−^ production in microglia, we investigated whether norfluoxetine altered NADPH oxidase by measuring the levels and localization of p47^phox^, which is one of the cytosolic components of NADPH oxidase. To test this, cortical cultures of rat microglia were pretreated with norfluoxetine (50 *μ*M) for 6 hours followed by lipopolysaccharide (100 *μ*g/ml) or vehicle (control) for 24 hours. Culture samples were separated into membrane and cytosolic components and examined by Western blotting. In LPS-treated microglia cultures, the levels of p47^phox^ protein were significantly increased in the membrane fraction (Figures 4(a) and 4(b); *p* < 0.01), indicating translocation and activation of this subunit. By contrast, in the norfluoxetine-treated culture samples, the levels of p47^phox^ protein were reduced in membrane fraction by 38% (Figures 4(a) and 4(b); *p* < 0.05) compared with vehicle-treated control. Norfluoxetine- (50 *μ*M) only-treated control had no effects on the levels of p47^phox^ protein in both the cytosolic and membrane fractions (Figures 4(a) and 4(b)).

To confirm the hypothesis that norfluoxetine inhibits microglial activation, leading to neuronal survival, we examined whether LPS-induced nitric oxide (NO) release by activated microglia could be affected by norfluoxetine. For this purpose, microglia cultures were pretreated with norfluoxetine (10-100 *μ*M) or fluoxetine (50-100 *μ*M) as a positive control [5] for 6 hours followed by LPS treatment for 24 hours. LPS significantly increased the concentration of nitrite (Figure 4(c), 31.7 ± 1.7 *μ*M; *p* < 0.001) in the media formed from NO in cortical microglia cultures compared to vehicle-treated control. By contrast, treatment with 50 *μ*M and 100 *μ*M norfluoxetine attenuated the levels of nitrite by 43% (Figure 4(c), 18.1 ± 1.5 *μ*M; *p* < 0.01) and by 53% (Figure 4(c), 15 ± 1.2 *μ*M; *p* < 0.001), respectively. 10 *μ*M norfluoxetine had no effects. Similarly, 100 *μ*M fluoxetine reduced the levels of nitrite by 32% (Figure 4(c), 21.6 ± 2.1 *μ*M; *p* < 0.01). 50 *μ*M fluoxetine had no effects.

## 4. Discussion

The present study is the first demonstration that norfluoxetine prevents degeneration of nigrostriatal DA neurons *in vivo* in MPTP-lesioned mice by inhibiting microglial activation and ROS production. Additional studies *in vitro* show that treatment with norfluoxetine suppresses NADPH oxidase activation and NO production in rat microglial cultures exposed to LPS.

Microglia are the resident macrophage-like cells of the central nervous system. Under pathological conditions, microglial activation is able to impact directly or indirectly on neuronal survival or death [6, 22, 25]. Microglia are known to transit from resting status to activated status, which can produce ROS and exacerbate loss of DA neurons in the PD patients and animal models of PD [6, 7, 26, 27]. Given the potential clinical significance of microglial activation and activated microglia-derived ROS in the PD [7, 27], many studies including ours have shown that blockade of microglial activation and ROS production is attributed to an increase of DA neuronal survival *in vivo* and *in vitro* [22, 23, 28, 29]. Our previous reports have shown that treatment with SSRIs, such as fluoxetine and paroxetine, attenuated MPTP- and LPS-induced microglial activation and expression of microglia-derived ROS in models of PD *in* vivo and *in vitro* [4, 5, 10]. These results are in line with the present data that norfluoxetine inhibits microglial activation and ROS production in MPTP-lesioned SN *in vivo*. Taken together, the present data suggest that the observed neuroprotection is associated with the ability of norfluoxetine to suppress microglial activation and ROS production in MPTP mouse model of PD.

Oxidative stress is a consequence of ROS overproduction, which is considered to be a crucial pathophysiological event in DA neuronal death in the PD [6, 7, 26]. ROS are unpaired electrons containing an oxygen molecule that is directly able to damage on DNA, protein, and lipids [2, 3]. Importantly, ROS are generated by NADPH oxidase complex that is composed of the cytosolic components (p47^phox^, p67^phox^, and Rac1) and the membrane components (gp91^phox^ and p22^phox^) [30]. Numerous studies, including ours, have demonstrated that O_2_^−^ and O_2_^−^-derived oxidants are produced by the activated microglia-derived NADPH oxidase and mediate the loss of nigral DA neurons in MPTP mouse models of PD [6, 7, 23, 24, 26]. MPTP-induced activation of NADPH oxidase was attenuated by fluoxetine and paroxetine, resulting in the survival of DA neurons [4, 5, 10]. In the present study, Western blot analysis showed that LPS induced NADPH oxidase activation as evidenced by an increased level of membrane translocation of p47^phox^ protein in cultures of rat microglia. Activation of LPS-induced NADPH oxidase was reduced by norfluoxetine, suggesting that inhibition of NADPH oxidase activation by norfluoxetine contributes to DA neuroprotection. This interpretation is supported by the finding that fluoxetine reduces NADPH oxidase activation and oxidative damages in the SN of LPS-lesioned rat and rescues DA neurons [10].

Accompanying microglial NADPH oxidase-derived ROS, accumulating experimental evidence demonstrates that microglial activation leads to the production of proinflammatory mediators, such as nitric oxide (NO), synthesized by iNOS *in vivo* and *in vitro* [23, 25, 26, 28]. NO-derived RNS may trigger intracellular death-related signaling pathways [31], leading to DA neuronal death [26–28, 32]. Our data revealed that norfluoxetine significantly reduced the LPS-induced NO production (measured by nitrite levels) in rat cortical microglia cultures. Much higher doses of fluoxetine than norfluoxetine also inhibits NO production in LPS-treated microglial cultures, indicating that norfluoxetine is more potent than fluoxetine. It is therefore likely that the observed neuroprotective effects of norfluoxetine are associated with inhibitory action of this compound on NO production in LPS-treated microglia.

Additionally, analysis of catecholamines (CAs) such as dopamine, norepinephrine, and epinephrine seems to be much better to understand norfluoxetine effects on nigrostriatal dopamine (DA) system. Regarding this, we have previously shown that treatment of fluoxetine prevents DA depletion of nigrostriatal DA system in the MPTP-treated mice [5]. Our unpublished observation revealed that in the MPTP-treated mice, fluoxetine restores the striatal levels of norepinephrine and epinephrine by 23.5% or 17.3%, respectively. As norfluoxetine, a pharmacologically active metabolite of fluoxetine, has the same neuroprotective properties on DA neurons as fluoxetine, we assume that norfluoxetine can exert similar effect on CAs as fluoxetine, which possibly results in partial restoration of CAs of nigrostriatal DA system in the MPTP-treated mice.

Taken together, these results suggest that SSRIs (fluoxetine and paroxetine) and their active metabolites and analogs may be beneficial for treating neurodegenerative diseases, such as PD, which are associated with microglia-derived oxidative stress.

## Data Availability

The data used to support the findings of this study are included within the article.

## References

[1] Beitz J. M. (2014). Parkinson’s disease: a review. *Frontiers in Bioscience*.

[2] Miller R. L., James-Kracke M., Sun G. Y., Sun A. Y. (2009). Oxidative and inflammatory pathways in Parkinson’s disease. *Neurochemical Research*.

[3] Zhao J., Yu S., Zheng Y., Yang H., Zhang J. (2017). Oxidative modification and its implications for the neurodegeneration of Parkinson’s disease. *Molecular Neurobiology*.

[4] Chung Y. C., Kim S. R., Jin B. K. (2010). Paroxetine prevents loss of nigrostriatal dopaminergic neurons by inhibiting brain inflammation and oxidative stress in an experimental model of Parkinson’s disease. *Journal of Immunology*.

[5] Chung Y. C., Kim S. R., Park J. Y. (2011). Fluoxetine prevents MPTP-induced loss of dopaminergic neurons by inhibiting microglial activation. *Neuropharmacology*.

[6] Chung Y. C., Ko H. W., Bok E. (2010). The role of neuroinflammation on the pathogenesis of Parkinson’s disease. *BMB Reports*.

[7] Wu D. C., Teismann P., Tieu K. (2003). NADPH oxidase mediates oxidative stress in the 1-methyl-4-phenyl-1,2,3,6-tetrahydropyridine model of Parkinson’s disease. *Proceedings of the National Academy of Sciences of the United States of America*.

[8] Hashioka S., Miyaoka T., Wake R., Furuya M., Horiguchi J. (2013). Glia: an important target for anti-inflammatory and antidepressant activity. *Current Drug Targets*.

[9] Leff-Gelman P., Mancilla-Herrera I., Flores-Ramos M. (2016). The immune system and the role of inflammation in perinatal depression. *Neuroscience Bulletin*.

[10] Chung E. S., Chung Y. C., Bok E. (2010). Fluoxetine prevents LPS-induced degeneration of nigral dopaminergic neurons by inhibiting microglia-mediated oxidative stress. *Brain Research*.

[11] Liu R. P., Zou M., Wang J. Y. (2014). Paroxetine ameliorates lipopolysaccharide-induced microglia activation via differential regulation of MAPK signaling. *Journal of Neuroinflammation*.

[12] Obuchowicz E., Bielecka A. M., Paul-Samojedny M., Pudelko A., Kowalski J. (2014). Imipramine and fluoxetine inhibit LPS-induced activation and affect morphology of microglial cells in the rat glial culture. *Pharmacological Reports*.

[13] Lee J. Y., Lee H. E., Kang S. R., Choi H. Y., Ryu J. H., Yune T. Y. (2014). Fluoxetine inhibits transient global ischemia-induced hippocampal neuronal death and memory impairment by preventing blood-brain barrier disruption. *Neuropharmacology*.

[14] Lim C. M., Kim S. W., Park J. Y., Kim C., Yoon S. H., Lee J. K. (2009). Fluoxetine affords robust neuroprotection in the postischemic brain via its anti-inflammatory effect. *Journal of Neuroscience Research*.

[15] Lee J. Y., Kim H. S., Choi H. Y., Oh T. H., Yune T. Y. (2012). Fluoxetine inhibits matrix metalloprotease activation and prevents disruption of blood-spinal cord barrier after spinal cord injury. *Brain*.

[16] Manolopoulos V. G., Ragia G., Alevizopoulos G. (2012). Pharmacokinetic interactions of selective serotonin reuptake inhibitors with other commonly prescribed drugs in the era of pharmacogenomics. *Drug Metabolism and Drug Interactions*.

[17] Unceta N., Ugarte A., Sanchez A., Gomez-Caballero A., Goicolea M. A., Barrio R. J. (2010). Development of a stir bar sorptive extraction based HPLC-FLD method for the quantification of serotonin reuptake inhibitors in plasma, urine and brain tissue samples. *Journal of Pharmaceutical and Biomedical Analysis*.

[18] Andres-Costa M. J., Proctor K., Sabatini M. T. (2017). Enantioselective transformation of fluoxetine in water and its ecotoxicological relevance. *Scientific Reports*.

[19] Lopez-Munoz F., Alamo C. (2013). Active metabolites as antidepressant drugs: the role of norquetiapine in the mechanism of action of quetiapine in the treatment of mood disorders. *Frontiers in Psychiatry*.

[20] Giulian D., Baker T. J. (1986). Characterization of ameboid microglia isolated from developing mammalian brain. *The Journal of Neuroscience*.

[21] Won S. Y., Kim S. R., Maeng S., Jin B. K. (2013). Interleukin-13/interleukin-4-induced oxidative stress contributes to death of prothrombinkringle-2 (pKr-2)-activated microglia. *Journal of Neuroimmunology*.

[22] Joers V., Tansey M. G., Mulas G., Carta A. R. (2017). Microglial phenotypes in Parkinson’s disease and animal models of the disease. *Progress in Neurobiology*.

[23] Chung Y. C., Baek J. Y., Kim S. R. (2017). Capsaicin prevents degeneration of dopamine neurons by inhibiting glial activation and oxidative stress in the MPTP model of Parkinson’s disease. *Experimental & Molecular Medicine*.

[24] Huh S. H., Chung Y. C., Piao Y. (2011). Ethyl pyruvate rescues nigrostriatal dopaminergic neurons by regulating glial activation in a mouse model of Parkinson’s disease. *Journal of Immunology*.

[25] Carniglia L., Ramirez D., Durand D. (2017). Neuropeptides and microglial activation in inflammation, pain, and neurodegenerative diseases. *Mediators of Inflammation*.

[26] Gao H. M., Liu B., Zhang W., Hong J. S. (2003). Critical role of microglial NADPH oxidase-derived free radicals in the in vitro MPTP model of Parkinson’s disease. *The FASEB Journal*.

[27] Liberatore G. T., Jackson-Lewis V., Vukosavic S. (1999). Inducible nitric oxide synthase stimulates dopaminergic neurodegeneration in the MPTP model of Parkinson disease. *Nature Medicine*.

[28] Bok E., Chung Y. C., Kim K. S., Baik H. H., Shin W. H., Jin B. K. (2018). Modulation of M1/M2 polarization by capsaicin contributes to the survival of dopaminergic neurons in the lipopolysaccharide-lesioned substantia nigra in vivo. *Experimental & Molecular Medicine*.

[29] L'Episcopo F., Tirolo C., Serapide M. F. (2018). Microglia polarization, gene-environment interactions and Wnt/*β*-catenin signaling: emerging roles of glia-neuron and glia-stem/neuroprogenitor crosstalk for dopaminergic neurorestoration in aged Parkinsonian brain. *Frontiers in Aging Neuroscience*.

[30] Cross A. R., Segal A. W. (2004). The NADPH oxidase of professional phagocytes--prototype of the NOX electron transport chain systems. *Biochimica et Biophysica Acta*.

[31] Brown G. C., Neher J. J. (2010). Inflammatory neurodegeneration and mechanisms of microglial killing of neurons. *Molecular Neurobiology*.

[32] Singh S., Das T., Ravindran A. (2005). Involvement of nitric oxide in neurodegeneration: a study on the experimental models of Parkinson’s disease. *Redox Report*.

